# Supplementary material for: Understanding high fructose corn syrup in popular snacks: Consumption, perceptions and labeling preferences
Source: PLoS One. 2026 Feb 25;21(2):e0341607. doi: 10.1371/journal.pone.0341607 (PMC12935215; doi:10.1371/journal.pone.0341607)
Supplement: S1 Table — This supporting information table reports the estimated coefficients, standard errors, and significance levels from the mixed logit specification used to evaluate consumer preferences. (DOCX) [file pone.0341607.s001.docx]

**Supporting information 1**

S1 Table. Mixed Logit Model Estimation

|  | Vanilla Yogurt | | Granola Bars | | Honey Wheat Bread | |
| --- | --- | --- | --- | --- | --- | --- |
| Attribute | Coefficient | | Coefficient | | Coefficient | |
| Does not contain HFCS | 0.999 | *** | 0.665 | *** | 0.592 | *** |
|  | (0.228) |  | (0.230) |  | (0.222) |  |
| Free of HFCS | 0.990 | *** | 0.991 | *** | 0.857 | *** |
|  | (0.181) |  | (0.195) |  | (0.172) |  |
| No HFCS | 0.008 |  | 0.775 |  | 0.236 |  |
|  | (0.216) |  | (1.095) |  | (0.294) |  |
| Contain HFCS | -0.637 | *** | -0.485 | ** | -0.908 | *** |
|  | (0.189) |  | (0.198) |  | (0.231) |  |
| Price | -0.809 | *** | -1.072 | *** | -0.848 | *** |
|  | (0.204) |  | (0.235) |  | (0.254) |  |
| ASC | -7.021 | *** | -8.372 | *** | -6.171 | *** |
|  | (0.777) |  | (0.987) |  | (0.695) |  |
| Standard Deviation | Coefficient | | Coefficient | | Coefficient | |
| Does not contain HFCS | 2.260 | *** | 2.578 | *** | 1.792 | *** |
|  | (0.450) |  | (0.494) |  | (0.427) |  |
| Free of HFCS | 1.048 | ** | 0.796 |  | 0.001 |  |
|  | (0.415) |  | (0.570) |  | (0.756) |  |
| No HFCS | 0.003 |  | 3.291 |  | 0.269 |  |
|  | (0.873) |  | (3.686) |  | (2.938) |  |
| Contain HFCS | 2.688 | *** | 3.294 | *** | 3.711 | *** |
|  | (0.501) |  | (0.579) |  | (0.592) |  |
| Price | 1.531 | *** | 1.834 | *** | 1.396 | *** |
|  | (0.187) |  | (0.251) |  | (0.169) |  |
| Observations | 6,780 | | 6,780 | | 6,780 | |
| Log Likelihood | -1831.792 | | -1813.4497 | | -1814.9981 | |
| Note: Standard errors in parentheses. ***indicates significance at 1%, ** indicates significance at 5%, and * indicates significance at 10%. No label in the product is used as baseline. ASC is the acronym of “Alternative Specific Constant” or the “None” option, and HFCS is the acronym of “High Fructose Corn Syrup”. | | | | | | |
