# Supplementary material for: Understanding high fructose corn syrup in popular snacks: Consumption, perceptions and labeling preferences
Source: PLoS One. 2026 Feb 25;21(2):e0341607. doi: 10.1371/journal.pone.0341607 (PMC12935215; doi:10.1371/journal.pone.0341607)
Supplement: S2 Table — This supporting information table presents results from the random-effects model linking sociodemographic characteristics with willingness to pay values. (DOCX) [file pone.0341607.s002.docx]

**Supporting information 2**

S2 Table. Random Effects Regression for sociodemographic characteristics

|  | Vanilla Yogurt | | Granola Bars | | Honey Wheat Bread | |
| --- | --- | --- | --- | --- | --- | --- |
| Attribute | Coefficient | | Coefficient | | Coefficient | |
| Age (Years) | 0.039 |  | -0.013 |  | 0.004 |  |
|  | (0.036) |  | (0.031) |  | (0.021) |  |
| College educated (1=Yes, 0=No) | -0.911 |  | -1.155 |  | -0.156 |  |
|  | (0.853) |  | (1.225) |  | (0.516) |  |
| Sex (1=Female, 0=Male) | -1.104 |  | -0.164 |  | 0.318 |  |
|  | (0.970) |  | (0.713) |  | (0.412) |  |
| Married (1=Yes, 0=No) | -1.640 |  | 0.732 |  | 0.451 |  |
|  | (1.904) |  | (0.999) |  | (0.547) |  |
| Race (White=1, Other=0) | 0.016 |  | -0.136 |  | 0.164 |  |
|  | (0.640) |  | (0.720) |  | (0.449) |  |
| Hispanic or Latino (1=Yes, 0=No) | 1.247 | * | 0.351 |  | -0.274 |  |
|  | (0.702) |  | (0.620) |  | (0.483) |  |
| Household size | -0.372 |  | 0.611 | * | -0.278 |  |
|  | (0.532) |  | (0.340) |  | (0.187) |  |
| Minors in household (1=Yes, 0=No) | 2.481 |  | -0.210 |  | 0.186 |  |
|  | (2.804) |  | (0.816) |  | (0.570) |  |
| Annual income (thousand USD) | 0.002 |  | 0.002 |  | 0.002 |  |
|  | (0.006) |  | (0.009) |  | (0.006) |  |
| Constant | 2.253 | ** | -2.968 |  | 2.110 | ** |
|  | (1.127) |  | (2.278) |  | (1.049) |  |
| Observations | 1,006 | | 1,006 | | 1,006 | |
| $\rho$ | 0.265 | | 0.149 | | 0.235 | |

Note: Random effects regression results showing the relationship between respondent characteristics and preference strength for high fructose corn syrup labeling across products. Standard errors in parentheses. *p < 0.10, **p < 0.05, ***p < 0.01. ρ represents the intra-class correlation
